# Effector Tregs: middle-men in TGFβ activation

**DOI:** 10.18632/oncotarget.4686

**Published:** 2015-06-27

**Authors:** John J. Worthington, Mark A. Travis

**Affiliations:** Manchester Immunology Group, Faculty of Life Sciences, University of Manchester, Manchester, UK

**Keywords:** immunology and Microbiology Section, Immune response, Immunity, regulatory T-cells, inflammatory bowel disease, integrins, transforming growth factor β

Regulatory T-cells (Tregs) are an inherent suppressive cell of the immune system with an established developmental requirement for the cytokine transforming growth factor β (TGFβ). However, the precise mechanisms by which mature Tregs utilize TGFβ during disease are unclear. In the May issue of Immunity, we have demonstrated that effector regulatory T-cells are essential activators of latent-TGFβ which is crucial to suppress ongoing inflammation.

A failure to regulate effective immunity results in chronic inflammation which can lead to immunopathology and carcinogenesis. One of the key molecules involved in immune cell suppression is the cytokine TGFβ, which crucially must be activated from its latent state in order to function [1]. An essential function of TGFβ is to drive the development of Tregs, both naturally derived thymic Tregs and the peripherally induced Tregs that are converted from the naïve T-cell population (pTregs) [2]. This critical subset of T-cells with an inherent suppressive role has been a huge focus of research both mechanistically and therapeutically, with current treatments for autoimmunity and transplantation involving the *ex vivo* expansion of patient's Tregs and conversely selective Treg depletion during cancer treatment.

Several *in vivo* studies have clearly shown that, in addition to its role in Treg development, TGFβ plays a fundamental role in the suppressive function of Tregs. However, the precise mechanisms by which TGFβ mediates Treg biology are unclear, with conflicting reports existing within the literature, most notably in studies utilizing T-cell transfer colitis. Within this colitis model, Tregs are essential for the suppression of disease but are still able to suppress inflammation when they lack the ability to produce TGFβ [3, 4]. However, the use of blocking antibodies in the same study demonstrates the complete dependence on TGFβ for disease suppression [3]. Furthermore, effector T-cells themselves must respond to TGFβ for Treg-mediated prevention of colitis, as T-cells require functional TGFβ receptors to be supressed [3]. Collectively, these data indicate that TGFβ is absolutely required for Tregs to suppress effector T-cells, but Tregs themselves do not need to be the source of TGFβ.

A particular focus within the Mark Travis lab at the Manchester Collaborative Centre for Inflammation Research, University of Manchester, is the regulation of latent TGFβ during intestinal inflammation. The TGFβ1 gene (TGFβ1 being the predominate isoform produced by the immune system) encodes latency associated peptide (LAP), which after transcription remains non-covalently bound preventing the active TGFβ dimer engaging its receptor. This so called LAP “straight-jacket” that surrounds the TGFβ dimer contains an RGD motif which can be bound by αv integrins allowing either conformational or protease dependent activation of latent TGFβ [1]. We have previously shown that tolerogenic CD103+ intestinal dendritic cells, which are key inducers of pTregs, are rich in the integrin αvβ8 and it is essential for their ability to activate latent TGFβ and convert naïve T-cells into pTregs [5]. A lack of this key regulatory molecule on DCs leads to an enhanced ability to fend off intestinal infection [6] but mice succumb to an age-related colitis [7]. We therefore postulated that, rather than produce TGFβ, Tregs may be required to activate the latent form to drive suppression.

We now demonstrate high levels of β8 integrin gene expression within the Treg population and utilising an active TGFβ reporter assay show that Tregs do indeed demonstrate an enhanced ability to activate latent TGFβ compared to other T-cell subsets. Furthermore, αvβ8 null Tregs lose their ability to activate latent TGFβ, suggesting that Treg cells activate enhanced levels of TGF-β versus other T cell subsets via expression of the integrin αvβ8.

Interestingly, we identified activated effector Tregs, thought to regulate ongoing inflammation, as the highest expressers of β8 integrin, indicating that this pathway maybe important in ongoing inflammation rather than homeostasis. As hypothesised, mice lacking β8 integrin expression specifically in Tregs (via Foxp3-cre), showed no overt autoimmune phenotype even after ageing, and Tregs lacking β8 integrin were capable of preventing the development of inflammatory T-cells in the intestine when co-transferred with effector T-cells in the transfer colitis model. Collectively, indicating TGF-β activation by Treg-cell-expressed integrin αvβ8 is not required for Treg-cell-mediated control of T cell tolerance at rest.

In order to examine the role during ongoing inflammation we returned to the T-cell transfer colitis model. In stark contrast to the co-transfer experiments, unlike control Tregs, Tregs that lacked β8 integrin expression completely lost their ability to cure colitis when transferred after effector T-cells had established ongoing inflammation. Moreover, when we examined the differing Treg and effector T-cell populations for downstream TGFβ signalling in the form of Smad2 phosphorylation, we saw that an increase in TGFβ signalling within the colitis-driving effector T-cell population correlated with suppression by Tregs. Importantly this increase was completely absent when the Tregs attempting to rescue colitis lacked β8 integrin expression, demonstrating that integrin αvβ8-mediated TGFβ activation by effector Tregs is essential for suppression of T-cell mediated inflammation. Finally, high expression of β8 integrin was also seen in human samples upon examination of the equivalent effector Treg populations.

This highlights a new suppressive mechanism by which Tregs control ongoing inflammation and is a pathway that can hopefully be targeted to prevent chronic inflammation, opening up the potential of therapy for a variety of inflammatory and autoimmune diseases via the manipulation of integrin αvβ8.

*“Integrin αvβ8-Mediated TGF-β Activation by Effector Regulatory T Cells Is Essential for Suppression of T-Cell-Mediated Inflammation”* was recently published in Immunity: 2015 May 19;42(5):903-15.
